# Editorial: Rising stars in urologic oncology: 2023

**DOI:** 10.3389/fruro.2025.1581141

**Published:** 2025-03-19

**Authors:** Juan Gomez Rivas

**Affiliations:** ^1^ Department of Urology, Health Research Institute, Hospital Clínico San Carlos, Madrid, Spain; ^2^ Facultad de Medicina, Universidad Complutense de Madrid, Madrid, Spain

**Keywords:** prostate, active surveillance, magnetic resonance imaging, microbiome, exosome

In the evolving landscape of urologic oncology, emerging researchers are crucial in pushing the boundaries of innovation, refining clinical practices, and enhancing patient outcomes. *Frontiers in Urology*’s *Rising Stars in Urologic Oncology: 2023* highlights some of the most promising contributions from early-career investigators, shedding light on key areas of advancement. This year’s Research Topic features four insightful studies that address contemporary challenges in prostate cancer and the role of the urinary microbiome in urological malignancies.

## Magnetic resonance imaging in active surveillance for prostate cancer

The management of prostate cancer has shifted towards a more personalized approach, with active surveillance gaining prominence as a viable strategy for men with low-risk disease. The study on *The Evolving Landscape: Magnetic Resonance Imaging in Active Surveillance for Prostate Cancer Management* explores the role of MRI in monitoring disease progression, aiming to improve patient selection and reduce unnecessary biopsies (De la Parra et al). With MRI’s increasing precision, its integration into surveillance protocols offers a promising path toward balancing effective cancer control with quality of life preservation

## The urinary microbiome and its potential role in urological cancers

The role of the human microbiome in oncogenesis has been a topic of growing interest, and this year’s Research Topic includes a mini-review titled *Urinary Microbiome and Urological Cancers* (Randazzo et al.). This review synthesizes current knowledge on how microbial composition in the urinary tract may influence the development and progression of urological malignancies, particularly bladder and prostate cancer. As the field moves toward personalized medicine, understanding the microbiome’s role may unlock new diagnostic and therapeutic avenues.

## Comparative analysis of primary prostate cancer treatment and metastatic disease progression

Despite advancements in localized prostate cancer treatment, a subset of patients progress to metastatic disease, raising critical questions about treatment sequencing and resistance mechanisms. The study *Comparative Analysis of Primary Prostate Cancer Treatment and Subsequent Metastatic Disease* provides valuable insights into treatment response patterns and disease trajectories (Shahait et al.). By identifying factors associated with metastatic progression, this research contributes to refining treatment strategies aimed at delaying or preventing disease spread.

## The emerging role of exosomes in prostate cancer

Exosomes have emerged as a novel component in the cancer microenvironment, with increasing evidence of their role in tumor progression, immune modulation, and potential as biomarkers. *Roles and Clinical Application of Exosomes in Prostate Cancer* delves into the intricate functions of these extracellular vesicles, exploring their diagnostic and therapeutic implications (Hu et al.). As liquid biopsy technologies advance, exosome-based approaches may offer minimally invasive tools for early detection, disease monitoring, and even targeted therapy.

## Conclusion: a new generation driving innovation

The *Rising Stars in Urologic Oncology: 2023* Research Topic exemplifies the forward-thinking research that is shaping the future of urologic oncology. By addressing key gaps in imaging, microbiome research, metastatic disease progression, and exosome biology, these studies contribute to a deeper understanding of cancer biology and patient management strategies. As these young investigators continue to make strides, their contributions will not only enhance scientific knowledge but also pave the way for more effective and personalized patient care in urological oncology.

Would you like any refinements or additional emphasis on particular themes?

